# Improved Gas Sensing Capabilities of MoS_2_/Diamond Heterostructures at Room Temperature

**DOI:** 10.1021/acsami.3c04438

**Published:** 2023-07-03

**Authors:** Michal Kočí, Tibor Izsák, Gabriel Vanko, Michaela Sojková, Jana Hrdá, Ondrej Szabó, Miroslav Husák, Karol Végsö, Marian Varga, Alexander Kromka

**Affiliations:** † Department of Semiconductors, Institute of Physics of the Czech Academy of Sciences, Cukrovarnická 10/112, Prague 6 162 00, Czech Republic; ‡ Department of Microelectronics, Faculty of Electrical Engineering, Czech Technical University in Prague, Technická 2, Prague 6 166 27, Czech Republic; § Department of Microelectronics and Sensors, Institute of Electrical Engineering, Slovak Academy of Sciences, Dúbravská Cesta 9, Bratislava 841 04, Slovak Republic; ∥ Department of Multilayers and Nanostructures, Institute of Physics, Slovak Academy of Sciences, Dúbravská Cesta 9, Bratislava 845 11, Slovak Republic; ⊥ Centre for Advanced Materials Application (CEMEA), Slovak Academy of Sciences, Dúbravská Cesta 5807/9, Bratislava 845 11, Slovak Republic

**Keywords:** gas sensors, H-terminated diamond, MoS_2_, MoS_2_/H-NCD heterostructure, room temperature, P−N junction, sensitivity, gas interaction model

## Abstract

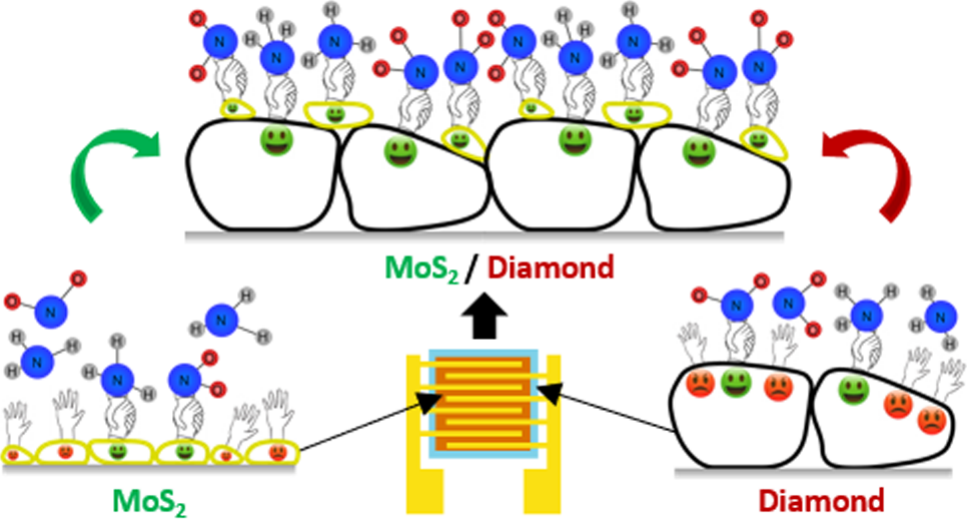

Molybdenum disulfide (MoS_2_) and nanocrystalline diamond (NCD) have attracted considerable attention due to their unique electronic structure and extraordinary physical and chemical properties in many applications, including sensor devices in gas sensing applications. Combining MoS_2_ and H-terminated NCD (H-NCD) in a heterostructure design can improve the sensing performance due to their mutual advantages. In this study, the synthesis of MoS_2_ and H-NCD thin films using appropriate physical/chemical deposition methods and their analysis in terms of gas sensing properties in their individual and combined forms are demonstrated. The sensitivity and time domain characteristics of the sensors were investigated for three gases: oxidizing NO_2_, reducing NH_3_, and neutral synthetic air. It was observed that the MoS_2_/H-NCD heterostructure-based gas sensor exhibits improved sensitivity to oxidizing NO_2_ (0.157%·ppm^–1^) and reducing NH_3_ (0.188%·ppm^–1^) gases compared to pure active materials (pure MoS_2_ achieves responses of 0.018%·ppm^–1^ for NO_2_ and −0.0072%·ppm^–1^ for NH_3_, respectively, and almost no response for pure H-NCD at room temperature). Different gas interaction model pathways were developed to describe the current flow mechanism through the sensing area with/without the heterostructure. The gas interaction model independently considers the influence of each material (chemisorption for MoS_2_ and surface doping mechanism for H-NCD) as well as the current flow mechanism through the formed P–N heterojunction.

## 1 Introduction

Gas sensors are essential for industry, healthcare, and almost everyday life, with an increasing emphasis on detecting hazardous substances and improving air quality.^1^ The development of sensors based on new materials with high sensitivity, stability, and reproducibility for the detection of various gases is therefore subject to high demands.^2−6^ Researchers are currently focusing on emerging two-dimensional (2D) materials, such as transition-metal dichalcogenides (TMDs), for use as active layers in gas sensing applications.

TMDs are a group of compounds with the chemical formula MX_2_, where M is a transition-metal atom and X is a chalcogen atom. Their structure consists of an atomic layer of transition metals sandwiched between two chalcogen layers.^7^ TMDs exhibit unique electronic structures and extraordinary physical and chemical properties for many applications.^3,7^ For example, TMDs are featured by a thickness-dependent electronic band structure,^8^ high charge carrier mobility,^9^ and in general a high surface-to-volume ratio, which is a natural asset for applications such as chemical sensors.^10^ Their properties, especially semiconductor properties, depend on the thickness of the layer; e.g., the band gap of MoS_2_ changes its value and type from direct (∼1.8 eV) to indirect (∼1.2 eV) as the number of layers increases.^7,11,12^ Therefore, TMDs could be bulk types, such as MoS_2_ grains or a film of nanoflakes. TMDs have several sensing applications.^11−13^ Although TMDs have excellent sensitivity at high temperatures (above 100 °C), bare layers have poor sensing properties at room temperature.^7^ Increasing temperature, UV illumination, or combination with other materials can improve these limitations as reported in the literature.^7,14^ For example, carbon-based materials,^15−17^ graphene,^18^ reduced graphene oxide,^2^ or metal oxides (ZnO_2_,^19^ SnO_2_,^20,21^ or TiO_2_^22^), have been proven to improve sensing characteristics. The NCD surface consists of sp^3^-hybridized carbon bonds that are chemically and mechanically stable. Surface-grafting specific atoms and functional chemical groups, such as oxygen, hydrogen, and amine groups, can tailor the wettability and influence the surface energy of NCD films, making the surface properties hydrophobic for hydrogen-terminated and hydrophilic for oxygen-terminated surfaces.^23^ It has already been shown that hydrogen-terminated nanocrystalline diamond (H-NCD) films, which exhibit P-type subsurface conductivity, reliably detect oxidizing and reducing gases.^24−26^

The solid-state resistive gas sensors can be manufactured from any material that reacts to the presence of gases.^1^ This type of sensor is most commonly used to detect oxidizing or reducing gases. Nowadays, gas sensors based on MO_X_ materials, which are heated to higher temperatures, are being studied intensively.^27^ The development of sensors working at room temperature is very demanding from the point of reduced consumption, reduced dimensions, and the possibility of use in hazardous areas. Among the carbon-based gas sensors, reduced graphene oxide (rGO)^28^ with a sensitivity of 0.004%·ppm^–1^^29^ and carbon nanotubes (CNTs)^30,31^ are mainly considered for gas sensors operating at room temperature. The second group that is intensively researched is represented by 2D materials. From this group, TMDs (MoS_2_, PtSe_2_, etc.)^2−7^ with a sensitivity of 0.3%·ppm^–1^ for MoS_2_ nanoworm films after 90 days at 150 °C^3^ and 2D MO_X_^27^ were presented. Furthermore, to improve the performance of the gas sensors, several strategies can be used. For example, in the case of carbon-based gas sensors, the performance was improved by fabricating heterostructures that consisted of carbon nanostructures with polymers,^29,32^ ceramic nanoparticles,^33^ or other suitable materials.

Similarly, mesoporous In_2_O_3_ nanocrystals for the detection of NO_X_ at room temperature have been recently published by Gao et al.^34^ Due to the synergistic effect between its mesoporous and highly crystalline nature, the detection limit from 1000 ppb to 100 ppm was achieved.^35^ Shaik et al.^36^ have introduced a NO_2_ sensor with a detection limit of 5 ppm at room temperature by using N-doped reduced graphene oxide (rGO). Moreover, the composites of carbon nanotubes combined with hexagonal WO_3_ are shown to detect low concentrations (100 ppb) of NO_2_ at room temperature.^37^

Here, we present a novel MoS_2_/H-NCD heterostructure as a prospective gas sensor with improved gas sensing parameters (response and recovery time) even at room temperature due to the synergistic effect of both materials. This improvement is compared and described within the proposed gas interaction model of the sensing principles of individual MoS_2_ and H-NCD materials and their heterostructure. The sensitivity and time domain characteristics of the sensors were investigated for two active gases: oxidizing NO_2_ and reducing NH_3_. They were chosen as representative gases largely produced by industries, worsening the air quality in the environment and hazardous to health in higher concentrations.^1,27^

## 2 Experimental Section

### 2.1 Active Layer Preparation

Thin MoS_2_ layers were prepared on three substrates—bare Si, SiO_2_/Si, and diamond-coated SiO_2_/Si (H-NCD/SiO_2_/Si). First, 4 in. SiO_2_/Si and Si wafers were ultrasonically cleaned in acetone, isopropyl alcohol, and deionized water for 10 min and dried by nitrogen flow. Subsequently, the MoS_2_ layers were prepared in a two-step process. In the first step, a 4 nm thin Mo layer was deposited using DC magnetron sputtering in an Ar atmosphere (10^–3^ mbar) from a Mo target at room temperature (about 22 °C). The DC power and emission current were 460 W and 0.3 A, respectively. The rotation speed of the sample holder controlled the thickness of the prepared Mo films. Next, the predeposited Mo layers were sulfurized in a custom-designed CVD chamber. The Mo layer was annealed in sulfur vapors at a high temperature of 800 °C in a N_2_ atmosphere at ambient pressure. The substrate was placed together with the sulfur powder in the center of the furnace so that the temperature of the substrate and the powder were the same during the growth,^38,39^ unlike the standard CVD method, which uses a two-zone furnace with different temperatures for the sulfur powder and the Mo substrate.

In the case of NCD film growth, a clean SiO_2_/Si wafer was first treated by applying ultrasonic agitation in a water-based diamond powder suspension (∼5 nm particles) for 40 min, followed by the growth in a linear antenna microwave plasma CVD system (Roth&Rau AK400) consisting of two linear antennas. The NCD was grown at a low deposition rate (about 15 nm/h) to a thickness of 450 nm (evaluated from the interference fringes of the reflectance spectra measured in the vis–NIR region). The process parameters of the linear antenna system are as follows: the power of the microwave generators was 2 kW, the pressure of the gas mixture was 0.15 mbar (200 sccm H_2_, 5 sccm CH_4_, and 20 sccm CO_2_), the deposition time was 30 h, and the substrate temperature was 550 °C. The surface of the as-grown NCD films was treated in hydrogen plasma to obtain hydrophobic properties. Surface functionalization by hydrogen was performed in a focused MW plasma CVD chamber (Aixtron P6 system, 1500 W, 30 mbar, 300 sccm of H_2_, 20 min, 500 °C). These layers are further referred to as H-NCD.

**Figure 1:**
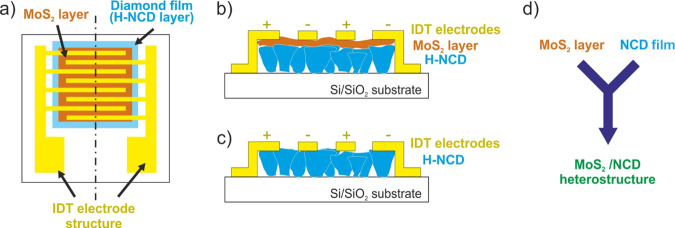
Schematic top (a) and cross-sectional views of MoS_2_/H-NCD/SiO_2_/Si (b), cross-sectional view of H-NCD/SiO_2_/Si (c) sensors, and schematic illustration of the combination of both materials in a heterostructure (d). In the case of the MoS_2_/SiO_2_/Si sensor, the MoS_2_ layer was prepared directly on the SiO_2_/Si substrate, and no diamond deposition was performed (not illustrated in this figure).

Finally, the deposited MoS_2_, H-NCD layers, and their heterostructure MoS_2_/H-NCD were coated with a 120 nm-thick Ti/Au (20 nm of Ti and 100 nm of Au) interdigitated electrode (IDT) structure for electrical connection on the top layer (Figure 1). The IDT was connected with measurement pins using a wire bonding technique for better electrical contact and handling. Metal contact pads were fabricated by a combination of electron beam evaporation and a consequent lift-off technique.

### 2.2 Characterization of MoS_2_ and Diamond Films

**Figure 2:**
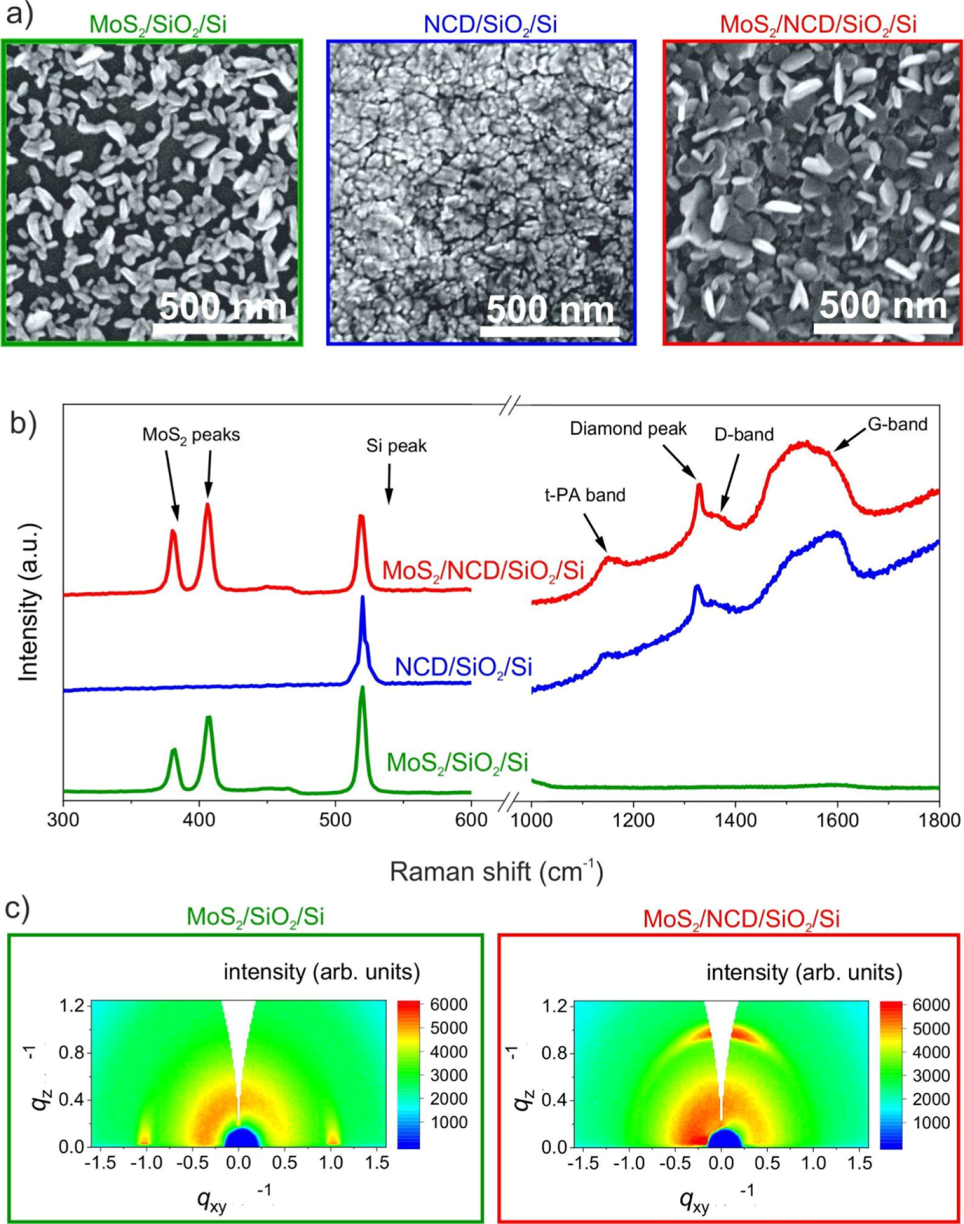
(a) Top-view SEM images of samples MoS_2_/SiO_2_/Si, H-NCD/SiO_2_/Si, and MoS_2_/H-NCD/SiO_2_/Si and (b) corresponding Raman spectra of samples taken at a 442 nm excitation wavelength; (c) GIWAXS reciprocal space maps of the MoS_2_/SiO_2_/Si and MoS_2_/H-NCD/SiO_2_/Si samples.

The surface morphology of the prepared samples was measured using a Tescan MAIA 3 scanning electron microscope at a 10 keV electron gun energy. The surface morphology of the samples is shown in Figure 2a. As shown in Figure 2a, the SiO_2_/Si substrate was covered with a completely closed H-NCD film. In contrast to SiO_2_, the surface coverage by MoS_2_ nanoflakes (flake size in the range of 50–100 nm) was lower for MoS_2_/H-NCD, probably due to the higher surface roughness. The MoS_2_ layer was also prepared on the reference Si substrate (the SEM image is shown in Figure S1 in the Supporting Information).

The chemical composition of the prepared samples was measured using a Renishaw inVia Reflex Confocal Raman microscope with a 442 nm excitation wavelength. As shown in Figure 2b, the H-NCD/SiO_2_/Si sample exhibits a typical Raman spectrum for NCD. In this spectrum, there is a representative peak of the Si substrate at 520 cm^–1^, a narrow peak at 1331 cm^–1^ attributed to the first-order diamond peak, and two broad bands labeled as D and G at 1350 and 1595 cm^–1^ and recognized as disordered sp^2^ carbon and graphitic phases, respectively.^24,26^ The MoS_2_/SiO_2_/Si sample is characterized by a Si peak at 520 cm^–1^ and two narrow peaks at 381 and 406 cm^–1^ attributed to MoS_2_.^7,40^ The Raman spectrum of MoS_2_/H-NCD/SiO_2_/Si combines all the peaks described above.

Grazing-incidence wide-angle X-ray scattering (GIWAXS) measurements were performed with a home-built system based on a microfocus X-ray source (Cu Kα, IμS, Incoatec) and a 2D X-ray detector (Pilatus 100K, Dectris). The angle of incidence on the sample was set to 0.2°. The sample–detector distance was 90 mm, as validated by a calibration standard (corundum). The collected GIWAXS patterns provided structural information about the prepared samples. Figure 2c shows reciprocal space maps of the as-prepared MoS_2_ films on the SiO_2_ and NCD films, respectively. The GIWAXS of the MoS_2_ film prepared on the reference Si substrate is shown in the Supporting Information (Figure S2a). The appearance of two symmetrical 002 diffraction spots at *q*_xy_ ∼ ±1 Å^–1^ for Si and SiO_2_/Si substrate means the vertical alignment of MoS_2_. It means that the *c*-axis is parallel to the substrate surface. Horizontal alignment was observed with the *c*-axis perpendicular to H-NCD/SiO_2_/Si, as confirmed by the position of the 002 diffractions at *q*_z_ ∼ 1 Å^–1^.

The wetting properties of the diamond film surfaces (H-termination and O-termination) were determined by contact angle measurements at room temperature using a static method in a material–water droplet system. The contact angle (wetting angle) was obtained by dropwise addition of a liquid onto the surface of a material. The surface tension of the liquid causes the drop to form a dome shape. 3 μL-volume water was added dropwise onto the diamond surface and captured by a digital CCD camera. The contact angles were calculated by a multipoint fitting of the drop profile using Surface Energy Evaluation software (Advex Instruments, Czechia). The H-terminated NCD is hydrophobic. A higher contact angle means more terminated hydrogen on the surface and thus a better response to the exposed gas. It should be noted that the optimal contact angle for a good H-termination is at least 90°.^26,39,41^ The contact angle of the prepared H-NCD/SiO_2_/Si samples was evaluated to be greater than 100°. The photographs of the measured contact angles are given in the Supporting Information (Table S1).

### 2.3 Experimental Setup for Gas Sensor Testing

A custom-built computer-controlled system was used for the characterization of the gas sensors. The creation of two independent gas mixtures (NH_3_ and NO_2_) with different concentrations and humidity is a major advantage of this experimental setup. The accuracy of this system is less than 1 ppm (measured by commercial gas sensors). However, the accuracy also depends on the purity of the delivered gases in the cylinder (the accuracy of the gas concentration in bottles is less than 0.1 ppm). The electrical characteristic (resistance change) was measured using a sensor holder with spring pins (Figure S3) and a source measure unit (SMU) Keithley SourceMeter 2401 with four-wire DC resistance measurement (Kelvin resistance measurement). The prepared sensors were measured with a voltage source with a nominal value of 0.1 V. A PC with a LabVIEW program was used to acquire the data from the SMU and ohmmeter. The four-input selection valve selects one input to the first output and three others to the second output (exhaust). The gas sensors were placed in the polycarbonate test chamber with two sections in series. The volume of one section was 22 cm^3^. The sensors were measured in the first section to minimize the time delay due to the gas exchanges in the chamber. The PT1000 sensor measures the temperature in the chamber throughout the measurement of the gas sensors. A photo of the experimental setup is shown in Figure S3.

## 3 Measurements and Results

Characterization of materials using SEM, Raman spectroscopy, and GIWAXS measurements is described in the previous chapter. In this part of the paper, we focus on the detailed characterization of the sensing properties of the prepared samples. First, the time-relative responses (i.e., response curves) of the fabricated conductivity gas sensors were measured for NH_3_ and NO_2_ gases at room temperature. The measured temperature was relatively stable, fluctuating between 21.8 and 22.5 °C, with an average of 22 °C. Active gases were used directly from gas bottles with the concentration and humidity defined and verified by the manufacturer (99.6 ppm in synthetic air (80% of N_2_ and 20% of O_2_) and <5% humidity for NO_2_ and 96.6 ppm in synthetic air and <5% humidity for NH_3_). In addition, 90% humid synthetic air without active gas was used at the end of the cycle to verify the effect of humidity on the sensors. The impact of increased humidity on the sensor’s response properties for NO_2_ and NH_3_ was not investigated. Gas humidity was measured with a commercial hydrometer at the same temperature as in the gas sensor measurements. Mixtures of active gases and synthetic dry air for measuring the response to different concentrations were used to create the appropriate concentration. The resistance change Δ_R_ was calculated by eq 1, where *R* represents resistance measured for selected gas and *R*_0_ is the initial resistance.
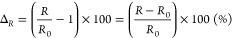
1

Four sensing layers were tested: reference H-NCD/SiO_2_/Si, reference MoS_2_/Si, MoS_2_/SiO_2_/Si, and heterostructure MoS_2_/H-NCD/SiO_2_/Si. The measured responses of the H-NCD/SiO_2_/Si reference sample are given in the Supporting Information (Chapter 3).

### 3.1 Gas Response of MoS_2_ on SiO_2_

**Figure 3:**
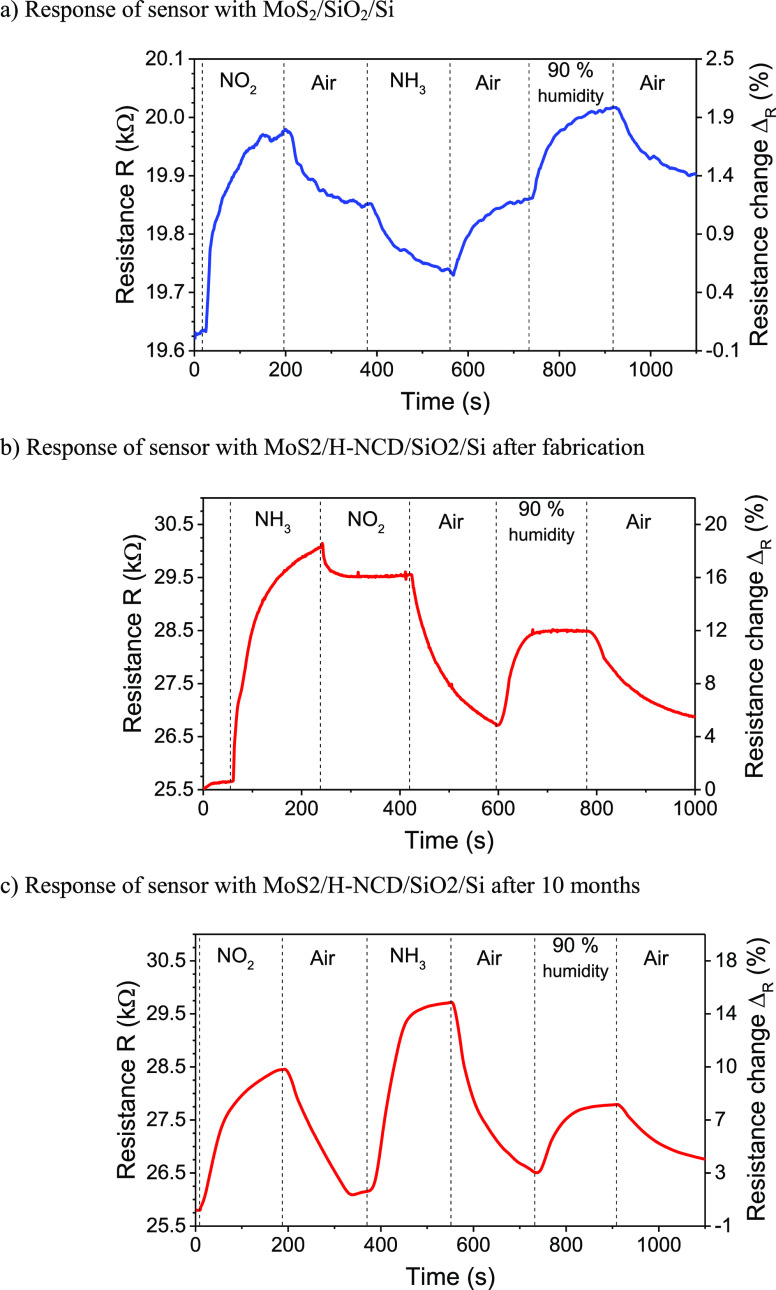
Time response of the sensor with MoS_2_/SiO_2_/Si (a), MoS_2_/H-NCD/SiO_2_/Si after fabrication (b), and MoS_2_/H-NCD/SiO_2_/Si after 10 months (c) to three gases (ammonia, nitrogen dioxide, and 90% humidity).

The first type of structure combines thin layers of MoS_2_ and SiO_2_. Figure 3a shows the absolute and relative change in resistance over time. The initial resistance (*R*_0_) is 19.6 kΩ, increasing by 1.8% to 20 kΩ for NO_2_. For NH_3_ the resistance decreases from 19.9 to 19.7 kΩ (−0.7%). From the measured gas responses, the calculated sensitivity of the MoS_2_/SiO_2_/Si sample is 0.018%·ppm^–1^ (3.53 Ω·ppm^–1^) for NO_2_ and −0.0072%·ppm^–1^ (1.41 Ω·ppm^–1^) for NH_3_.

### 3.2 Gas Response of MoS_2_ on Diamond

The time response of the MoS_2_/H-NCD heterostructure on the SiO_2_/Si substrate to three gases was measured at room temperature (22 °C) as in the previous measurement. Figure 3b shows the absolute and relative change in resistance over time. The resistance increases by 17.8% from 25.5 to 30 kΩ for NH_3_. This value is more than 25 times higher than that for MoS_2_/SiO_2_/Si. After this, NO_2_ is released into the test chamber. The resistance changes the value to 29.5 kΩ, and the percentual change is 15.7%. This value is approximately 9 times higher than that of the MoS_2_/SiO_2_/Si sample. The calculated sensitivity is 0.1884%·ppm^–1^ (48 Ω·ppm^–1^) for NH_3_ and 0.1572%·ppm^–1^ (40 Ω·ppm^–1^) for NO_2_.

Figure 3c shows the absolute and relative change in resistance over time measured after 10 months of sample storage in air. This measurement examined the time stability (i.e., aging) of the heterostructure to NH_3_ and NO_2_. The response decreases by only 2.6% for NH_3_ and by 5.2% for NO_2_. The average monthly fluctuations of the gas responses are 0.26%·months^–1^ for NH_3_ and 0.52%·months^–1^ for NO_2_.

### 3.3 Comparison of Sensors

**Figure 4:**
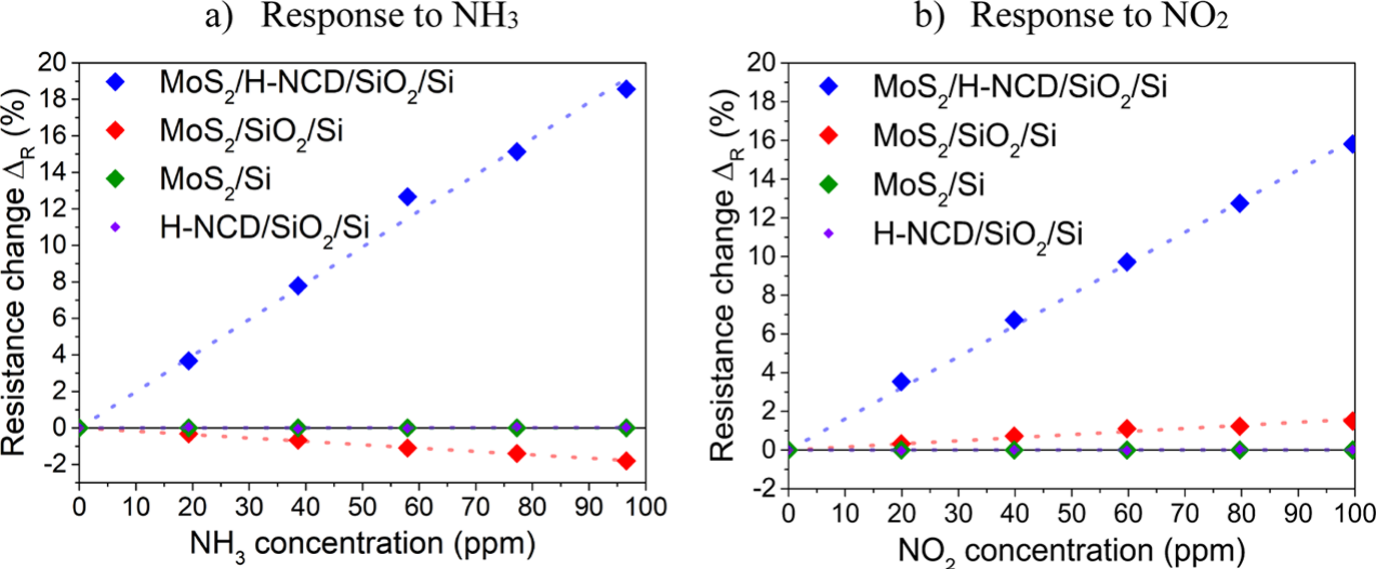
Relative resistance change of sensors with MoS_2_/H-NCD/SiO_2_/Si, MoS_2_/SiO_2_/Si, MoS_2_/Si, and H-NCD/SiO_2_/Si to six different concentrations of ammonia (a) and nitrogen dioxide (b).

A comparison of the relative changes in resistance of all sensor types is plotted in Figure 4a for different NH_3_ concentrations and in Figure 4b for NO_2_ at room temperature (22 °C). The Δ_R_ value (i.e., the response) increases/decreases linearly with an active gas concentration in all cases. The values have a small deviation (max. 1.8%) from linear interpolation.

The electronic characteristics and responses for all sensors are summarized in Table 1. The table includes the measured data for all sensors. It can be concluded that the MoS_2_/Si and H-NCD/SiO_2_/Si samples are not suitable for gas sensing at room temperature. The MoS_2_/SiO_2_/Si sample slightly increased the gas response and the initial resistance. However, the resistance change of the active layer is still low. The MoS_2_/H-NCD/SiO_2_/Si structure does not increase the initial resistance but improves the gas response on the active layer. Compared to previous types of sensors, MoS_2_/H-NCD/SiO_2_/Si exhibited improved resistance change for both oxidizing and reducing gases. Unfortunately, this heterostructure has lost its selectivity for the recognition of oxidizing and reducing gases as it increases resistance to both types of gas. For the MoS_2_/H-NCD/SiO_2_/Si heterostructure, the minimal detection concentration for the change of 1% is 7 ppm and 5 ppm for NO_2_ and NH_3_, respectively.

**Table 1:** Comparison of Response and Characteristics of Different Sensor Types

	MoS_2_ on Si	diamond on SiO_2_	MoS_2_ on SiO_2_	MoS_2_ on diamond	
				at 0 day	after 10 months
*R*_0_ (kΩ) (source: 0.1 V)	0.006	17.8	19.6	25.5	25.8
(*R* – *R*_0_)·*R*_0_^–1^ response to 96.6 ppm NH_3_ (%)	<0.01	<0.01	–0.7	17.8	15.2
(*R* – *R*_0_)·*R*_0_^–1^ response to 99.6 ppm NO_2_ (%)	<0.01	<0.01	1.8	15.7	10.5
time response to 96.6 ppm NH_3_ (Ω·s^–^^1^)	0	0	–3.18	179	103
time response to 99.6 ppm NO_2_ (Ω·s^–^^1^)	0	0	9.26	181	63
sensitivity to NH_3_ (%·ppm^–^^1^)	<0.0001	<0.0001	–0.0072	0.1884	0.1573
sensitivity to NO_2_ (%·ppm^–^^1^)	<0.0001	<0.0001	0.0180	0.1572	0.1054

## 4 Discussion

Experimental gas sensing measurements show that the MoS_2_/H-NCD/SiO_2_/Si heterostructure is fully functional and enhances the gas sensing characteristics at room temperature. Both materials exhibit different types of conductivity. The MoS_2_ nanoflakes represent an N-type semiconductor (excess negative charge carriers), and the H-NCD forms a two-dimensional subsurface hole gas (2DHG) with P-type conductivity (excess positive charge carriers). Different conductivity types cause opposite responses (and reactions) when exposed to reducing and oxidizing gases. The following subsections describe the interaction of gas molecules at the active layers of the fabricated sensors.

### 4.1 Gas Interaction Model

**Figure 5:**
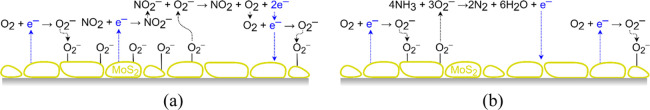
Schematic illustration of the gas sensing mechanism between a layer of MoS_2_ nanoflakes and (a) oxidizing and (b) reducing gases.

MoS_2_ generally behaves as an N-type semiconductor.^7^ The change in resistance in MoS_2_ nanoflakes is caused by chemisorption, reflecting the sorption of oxygen molecules on its solid surface by chemical bonding with electron transfer. Defects in MoS_2_, such as flake edges and sulfur vacancies, serve as active sites for the gas molecules under investigation. Gas sensing properties depend on the charge transfer between the gas molecules and defects in MoS_2_.^2,3,40^Figure 5 shows a schematic illustration of the gas sensing mechanism based on already published works.^2−5^ First, the O_2_ molecule from the air chemisorbs to the surface of MoS_2_ and forms a native oxide. These molecules act as electron trap centers, extracting electrons from MoS_2_ and generating O_2_^–^.^2,3^ As a result, the concentration of free electrons decreases, and consequently, the conductivity decreases too. The chemisorbed oxygen sets the baseline resistance of the sensing layer. For the oxidizing gas NO_2_ (Figure 5a), the gas molecules form NO_2_^–^ ions,^2−4^ which increase the resistivity of the layer. After switching the oxidizing gas to synthetic air, NO_2_^–^ ions react with chemisorbed O_2_^–^ to form NO_2_ and O_2_. The two remaining electrons from the chemical reaction are released back into the conduction band of MoS_2_ or form new O_2_^–^ ions with O_2_.^2,4,6,22,40^ On the other hand, the reducing gas NH_3_ (Figure 5b) reacts with chemisorbed O_2_^–^ ions and creates H_2_O and N_2_.^5^ The remaining electron from the reaction is released into MoS_2_ and reduces the resistivity of the sensing layer.^3,20^ During the recovery process, i.e., after the change of the reducing gas to synthetic air, O_2_ is chemisorbed from the atmosphere onto the surface of MoS_2_.^5,6,14,20,40^

On the other hand, H-NCD reveals unique properties of P-type induced subsurface conductivity, also known as 2DHG, which is sensitive to exposed gas or organic molecules.^25,26^ The change in resistance of H-NCD is caused by chemical reactions forming counterions on its surface via the electron transfer model.^25^ The gas interaction model with the widely established H-NCD subsurface doping mechanism is described in ref.^41^ The water molecule from the air humidity dissociates the ions H_3_O^+^ and OH^–^. The H_3_O^+^ ions attract electrons from the diamond surface, leading to P-type subsurface conductivity.

**Figure 6:**
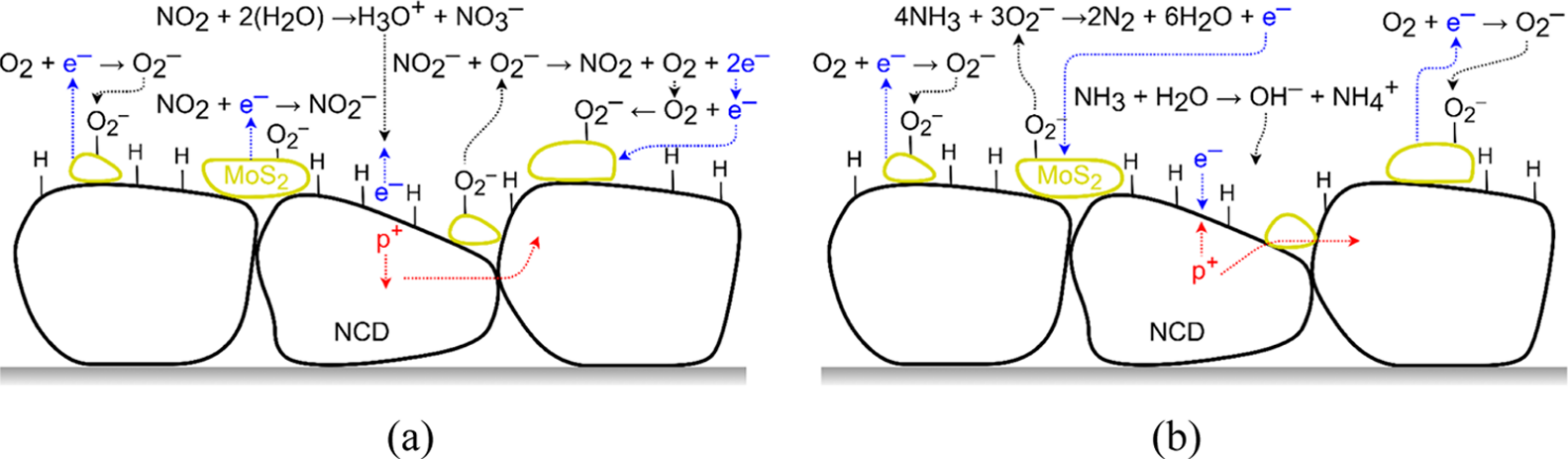
Schematic illustration of the gas sensing mechanism and charge transport for two parallel connected layers represented by MoS_2_ nanoflakes and H-NCD exposed to the (a) oxidizing and (b) reducing gas.

Thus, the MoS_2_/H-NCD/SiO_2_/Si heterostructure shows two types of conductivity: P-type H-NCD^26^ and N-type MoS_2_.^3^ This combination provides a unique material platform in which different conductivity types react oppositely to reducing and oxidizing gases.^17^ The gas interaction could be influenced by several factors, such as surface-controlled charge injection into/out of the depletion region, surface shortcuts from diamond or MoS_2_ layers, modulation of the P-type diamond subsurface conductivity by MoS_2_ (the gating-like effect), and the gradual degradation of the P-type diamond subsurface conductivity due to the deposition of MoS_2_ and others. Although the primary origin is still under investigation, the simplified model should be based on the coupling of two conduction paths via H-NCD or MoS_2_ layers. The change in resistance of the MoS_2_/H-NCD sensor is caused by (1) chemical reactions forming counterions on H-NCD and (2) chemisorption of oxygen molecules on the solid surface of MoS_2_ by chemical bonding with electron transfer. Its gas-sensing properties further depend on the charge carrier concentrations for both materials. Figure 6 gives a schematic illustration of the gas sensing mechanism for two layers coupled in parallel. Suppose there are oxidizing gas molecules in their vicinity (Figure 6a). In this case, the number of charge carriers increases for H-NCD and decreases for MoS_2_. Thus, the charge carrier transport mainly prevails through the diamond layer rather than through the MoS_2_ layer, while this charge carrier transport is scattered at the diamond grain boundaries. The reducing gas (Figure 6b) causes a decrease in the number of charge carriers for H-NCD and an increase for MoS_2_. As a result, the resistance of the MoS_2_ nanoflakes decreases and more charge carriers flow through these nanoflakes with lower resistance than through the potential barriers between individual diamond grains. However, H-NCD blocks the final charge transport due to its total area coverage. The total resistance is therefore higher for NH_3_ than for NO_2_ because the surface coverage of the MoS_2_ nanoflakes is low and H-NCD has a more pronounced effect on the change in resistance for reducing gases.

**Figure 7:**
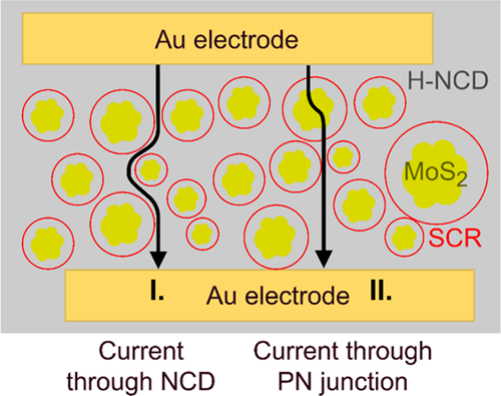
Schematic illustration of two ways (I and II) for the current flow between IDT electrodes, I—horizontal flow through H-NCD and II—combined horizontal/vertical flow, i.e., horizontal through H-NCD and MoS_2_ and vertical through the MoS_2_/H-NCD heterostructure.

Reducing and oxidizing gases contribute to the increased resistance of the MoS_2_/H-NCD heterostructure. In addition to the mechanisms described above, two effects of the resistance change are also manifested. The current flow and the subsequent resistance change consist of mutually constrained components:I. the horizontal one representing the current through the H-NCD and II. the vertical one representing the current through the MoS_2_/H-NCD. The schematic illustration is shown in Figure 7. The current flowing through the P–N junction must tunnel through the space charge region (SCR). When the gas is applied, the width of the SCR (w_SCR_) increases, and thus, the resistance increases too. The w_SCR_ can be calculated from the concentrations of free charge carriers injected into the semiconductors by the gases according to formula 2. The concentration of free charge carriers in H-NCD (N_A_) increases for the oxidizing gas (NO_2_) and decreases for the reducing gas (NH_3_), as described in the previous model. For N-type MoS_2_, the concentration has the opposite effect. So, the concentration (*N*_D_) decreases for NO_2_ and increases for NH_3_. The formula shows that the SCR width increases for both types of gas. As the width of the SCR increases, the number of charge carriers tunneling through the SCR decreases; thus, the total resistance is increased. In the case of the current flowing through H-NCD, formula 3 can be considered. The deposited interdigital electrodes measure this current component, while the additional resistance includes the distance between adjacent fingers. In the presence of the oxidizing or reducing gas, the geometric dimensions of the 2DHG change due to the increase in the width of the SCR, and thus, the total length *l* is increased, and the cross-section *S* is reduced. The total resistance therefore increases.
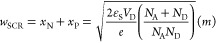
2
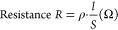
3

In addition, to support the importance of our model, we also investigated the role of H-NCD in the MoS_2_/H-NCD/SiO_2_/Si sensor. The measured responses and contact angles are given in the Supporting Information (Tables S1 and S2).

### 4.2 Effect of Oxidizing vs Reducing Gases

**Figure 8:**
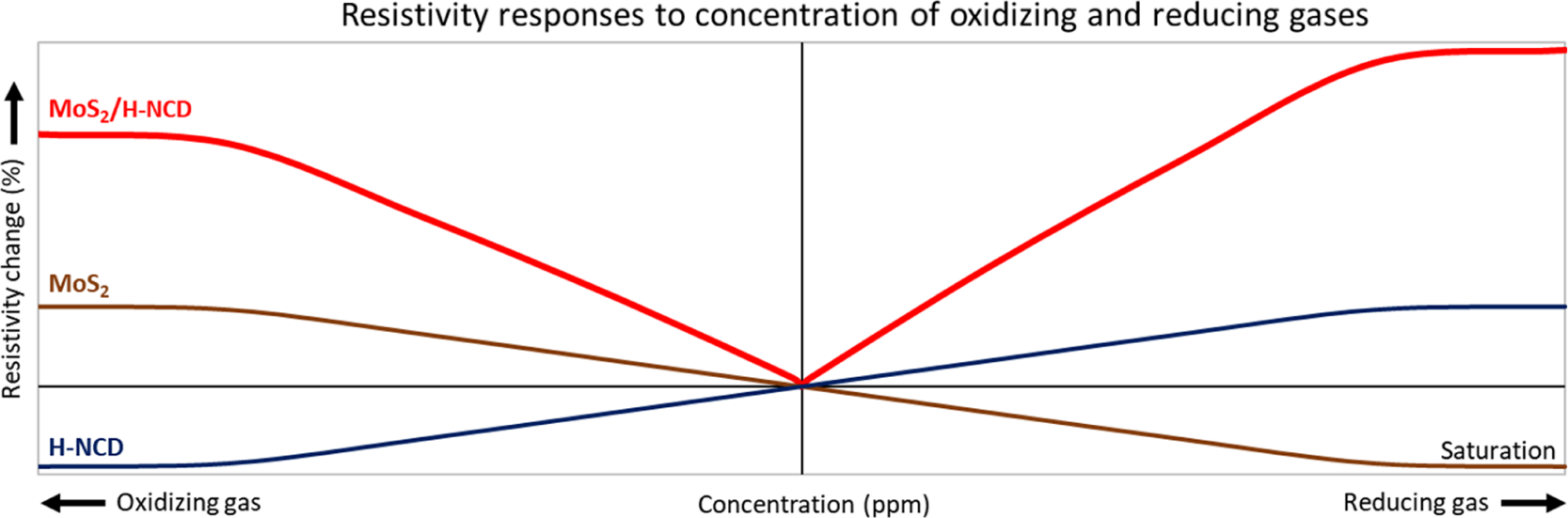
Qualitative illustration of the relative responses of MoS_2_, H-NCD, and MoS_2_/H-NCD sensor devices to different concentrations of oxidizing and reducing gases based on gas interaction models.

As described above, individual MoS_2_ and hydrogen-terminated diamonds are capable of recognizing oxidizing/reducing gases but with opposite signs of resistance change as illustrated in Figure 8. Here, the *Y*-axis represents only qualitative information and not quantitative. Unfortunately, the H-NCD did not reveal any response to exposed gases at room temperature, but the illustrative behavior was achieved for temperatures higher than 40 °C (see S5), which is in good agreement with our previous work.^41^ The MoS_2_/H-NCD heterostructure has a different response to gases as it increases resistance to both types of gases (i.e., it loses selectivity to oxidizing/reducing gas). The magnitude of the change also depends on the gas type; i.e., it is lower for the oxidizing gas than for the reducing gas, which can be attributed to the dominance of H-NCD in the MoS_2_/H-NCD heterostructure. However, heterostructures prepared with different ratios of diamond to MoS_2_ can further tailor the response to oxidizing and reducing gases.

## 5 Conclusions

MoS_2_/Si, MoS_2_/SiO_2_/Si, H-NCD/SiO_2_/Si, and MoS_2_/H-NCD/SiO_2_/Si structures were used to fabricate conductivity gas sensors and tested at room temperature (22 °C). The active layers of MoS_2_ and H-NCD were analyzed by SEM, Raman spectroscopy, contact angle, and GIWAXS measurements in their individual and combined forms. In terms of gas sensing properties, MoS_2_ and H-NCD showed poor responses at room temperature. However, by combining them into a MoS_2_/H-NCD heterostructure, the gas sensing parameters were significantly improved. The formed heterostructure, consisting of the P-type subsurface conductive H-NCD layer and the N-type conductive MoS_2_ nanoflakes, resulted in a synergistic effect that enhanced the gas response. While well-established interactions of gas molecules were experimentally validated for the particular form of MoS_2_ and H-NCD layers, the MoS_2_/H-NCD heterostructure did not reveal such a specific behavior. The presented model pointed out the influence of the P–N junction, especially the geometrical variation of the SCR, after its exposure to the tested gases. Unfortunately, this heterostructure abolishes the selectivity; i.e., increased resistance was observed for oxidizing and reducing gases with different responses. However, the combination of a MoS_2_/H-NCD heterostructure with a single MoS_2_ layer within one sensor chip seems to be a promising solution to overcome this limitation. This sensor can select the gas type on the MoS_2_ according to a mark of resistance change and the gas concentration by the size resistance change of the MoS_2_/H-NCD. In conclusion, this article introduces a new class of conductivity gas sensors that can provide miniaturization and reduction of power consumption compared to commercial sensors. The presented TMD/diamond heterostructures could be very suitable for portable devices or energy-harvesting applications.

## Supporting Information Available

Surface morphology of all samples, photo of the sensor testing setup, gas responses of reference samples, verification of the synergy effect between MoS_2_ and H-NCD via the O-term NCD, and water contact angle measurements (PDF)
