# Supplementary material for: Improved Gas Sensing Capabilities of MoS2/Diamond Heterostructures at Room Temperature
Source: ACS Appl Mater Interfaces. 2023 Jul 3;15(28):34206–14. doi: 10.1021/acsami.3c04438 (PMC10360066; doi:10.1021/acsami.3c04438)
Supplement: Supplementary file 1 — am3c04438_si_001.pdf [file am3c04438_si_001.pdf]

Supplementary information to: **Improved Gas Sensing Capabilities of MoS_2 / Diamond Heterostructures at Room Temperature**

Michal Kočí[1,2,*], Tibor Izsák[3], Gabriel Vanko[3], Michaela Sojková[3], Jana Hrdá[3], Ondrej Szabó[1], Miroslav Husák[2], Karol Végsö[4,5], Marian Varga[1,3], Alexander Kromka[1]

[1] Department of Semiconductors, Institute of Physics of the Czech Academy of Sciences, Cukrovarnická 10/112, Prague 6 162 00, Czech Republic

[2] Department of Microelectronics, Faculty of Electrical Engineering, Czech Technical University in Prague, Technická 2, Prague 6, 166 27, Czech Republic

[3] Department of Microelectronics and Sensors, Institute of Electrical Engineering, Slovak Academy of Sciences, Dúbravská Cesta 9, Bratislava, 841 04, Slovak Republic

[4] Department of Multilayers and Nanostructures, Institute of Physics, Slovak Academy of Sciences, Dúbravská Cesta 9, Bratislava, 845 11, Slovak Republic

[5] Centre for Advanced Materials Application (CEMEA), Slovak Academy of Sciences, Dúbravská Cesta 5807/9, Bratislava, 845 11, Slovak Republic

*Corresponding author e-mail: kocim@fzu.cz, kocimic1@fel.cvut.cz

1. SEM images and GIWAXS maps

The MoS_2 layer was prepared on three different substrates: Si, SiO_2/Si, and diamond-coated SiO_2 /Si (H-NCD/SiO_2 /Si). The surface morphology of the samples is compared in Figure S1 together with bare Si and bare SiO_2/Si samples. In addition, Figure S2 compares the GIWAXS reciprocal space maps of the MoS_2/Si and MoS_2/SiO_2/Si samples.

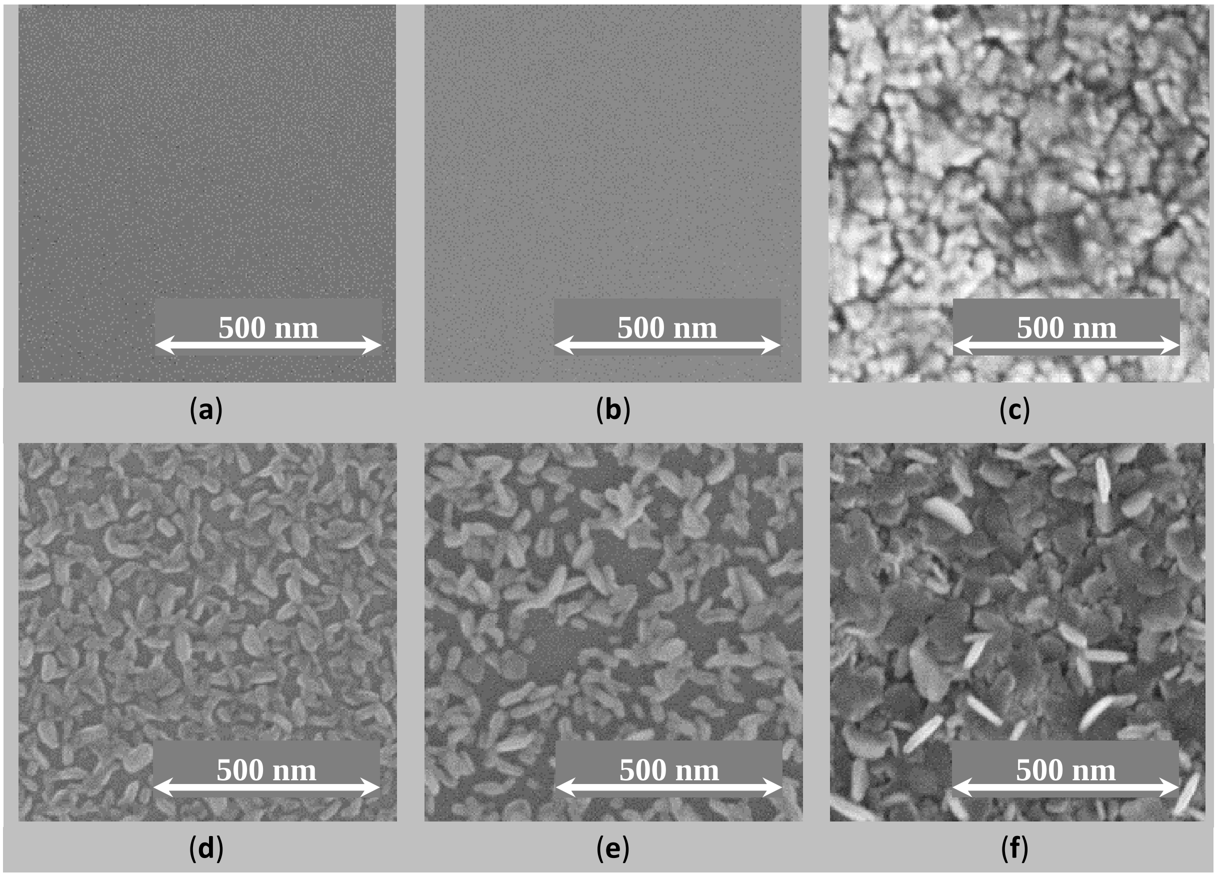

Figure S1 SEM images of different samples: (a) bare Si, (b) SiO_2/Si, (c) H-NCD/SiO_2/Si, (d) MoS_2/Si, (e) MoS_2/SiO_2/Si and (f) MoS_2/H-NCD/SiO_2/Si.

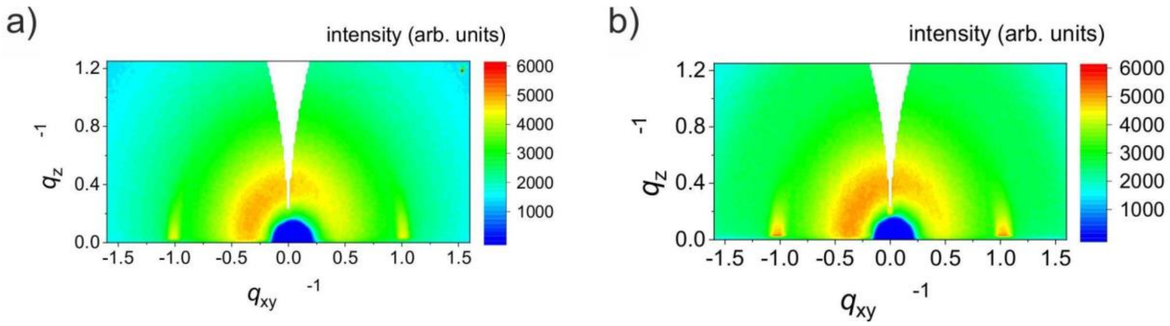

Figure S2 Comparison of GIWAXS reciprocal space maps of the (a) MoS_2/Si and (b) MoS_2/SiO_2/Si samples.

2. Gas sensor testing setup

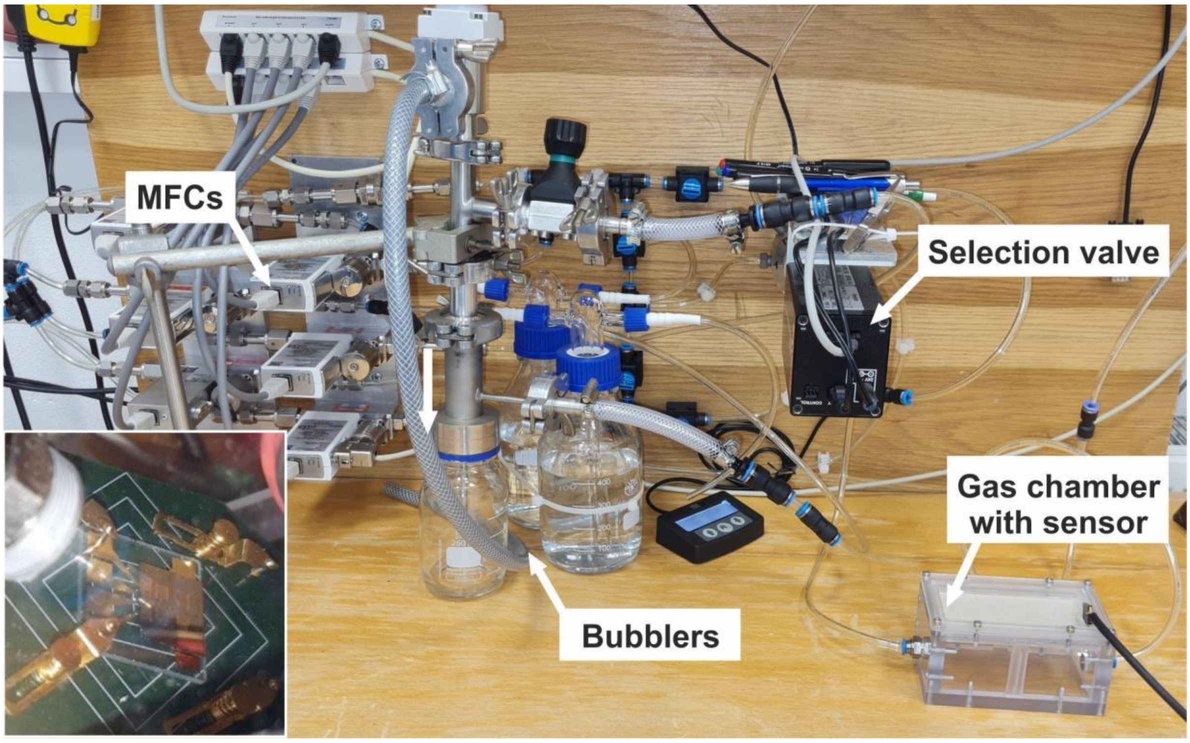

Figure S3 Photo of the experimental setup for gas sensor testing. Inset: wire-bonded sensor in the gas chamber.

3. Gas responses of reference samples

The time response of MoS_2 and H-NCD/SiO_2 conductivity gas sensors on Si substrate to three different types of gas (oxidizing, reducing and synthetic air) was measured in a test chamber at room temperature (about 22 °C). The measured enduring resistance of the MoS_2/Si was as low as 0.6 Ω. The absence of the insulating layer between the MoS_2 and the Si substrate causes low resistance, while silicon represents a shortcut. The steady-state value of the NCD/SiO_2/Si sample was measured at 17.8 kΩ.

The MoS_2 and NCD/SiO_2/Si impedance-based sensors responded poorly to the reducing (NH_3) and oxidizing (NO_2) gases at low temperatures. The NCD/SiO_2/Si only responds to 90 % humidity by changing its resistance. It can be therefore concluded that MoS_2/Si is unsuitable as a gas sensing layer, but the growth of MoS_2 was well-optimized on Si substrates as a reference.

3.1 Gas response of the H-NCD layer

The second sample that was tested was the NCD film grown on the SiO_2/Si substrate. Figure S4 shows the change in resistance and its percentage change dependence over time as a function of different gases. Unlike MoS_2, the bulk bare NCD layer is an insulator. However, it could exhibit P-type subsurface conductivity if hydrogen atoms sufficiently terminate its surface. The steady-state value of the resistance was measured at 17.8 kΩ. The gas response of the NCD has a low value due to the low temperature. The NCD/SiO_2/Si sample, similar to the

MoS$_2$/Si sample, only reacts to 90 % humidity, reducing the resistance by 17 %. Therefore, the prepared NCD sample is unsuitable for gas sensing applications at room temperature. The response to gases in H-NCD is caused by a chemical reaction forming counter-ions on its surface via the electron transfer model. At room temperature, the chemical reaction of a pure material is not supported by higher temperature or a chemical catalyst. For this reason, the chemical reaction is minimal, and the response is immeasurable. It is necessary to use higher temperatures or another material as a catalyst to increase the gas response. The responses at 40 °C are shown in Figure S5 for a) reducing gas ammonia and b) oxidizing gas nitrogen dioxide. The responses equal to theoretical gas interaction model at higher temperature.

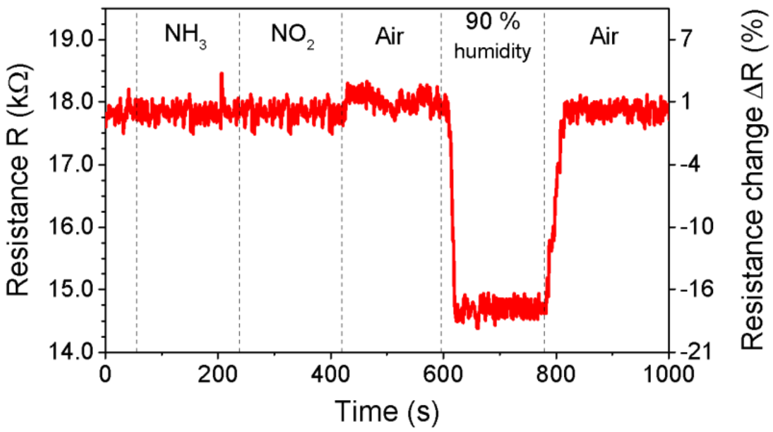

Figure S4 Time response of H-NCD/SiO$_2$/Si to three types of gases (ammonia, nitrogen dioxide, and synthetic air with 90 % humidity).

a)

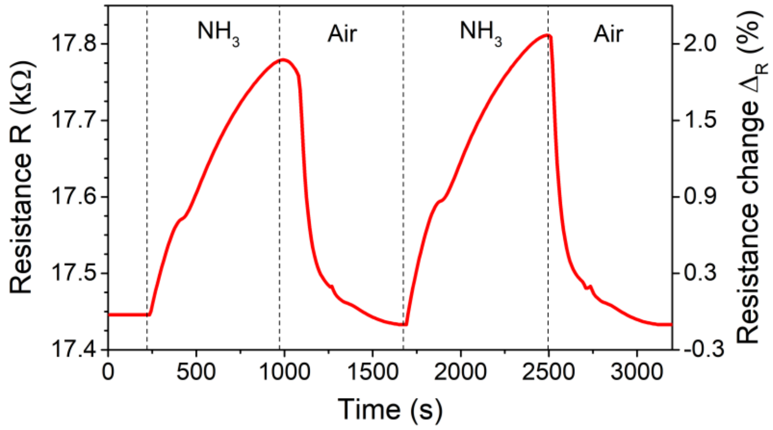

b)

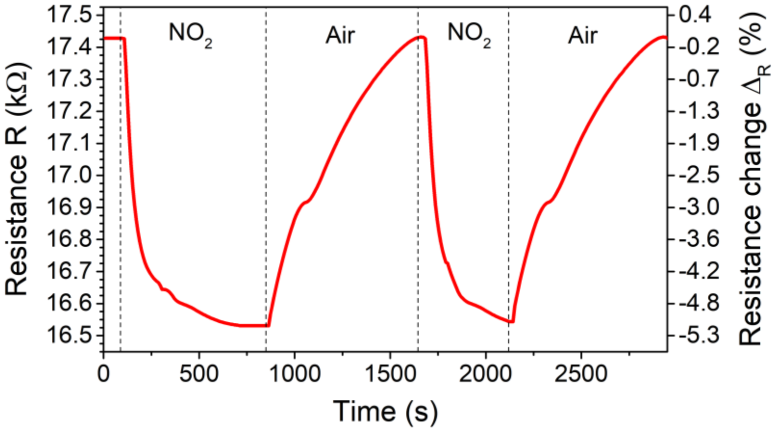

Figure S5 Time response of H-NCD/SiO_2/Si at 40 °C to a) ammonia NH_3 and b) nitrogen dioxide NO_2

4. Verification of synergy effect between MoS2 and H-NCD

The measurement of the water contact angle indirectly verifies the quality of the hydrogen termination. The H-NCD is hydrophobic. A higher contact angle means more terminated hydrogen on the surface and, thus, a better response to the exposed gas. The minimal contact angle for good sensing properties is about 90 °. The fabricated layers revealed similar contact angles over 100 °.

The MoS_2/NCD/SiO_2/Si sample was modified to verify the synergistic effect between the H-NCD and MoS_2 nanoflake layers. Firstly, the contact angles and responses of the MoS_2/NCD/SiO_2/Si and NCD reference layers were measured. Subsequently, the samples were placed in oxygen plasma to replace H-termination by O-termination, which does not reveal the 2DHG subsurface conductivity. The surface functionalization by oxygen was performed in a MW O_2 plasma chamber (Tesla system, 100W, 60 Pa, 50 sccm of H_2, 4 min). These layers will be further referred to as O-NCD layers. The contact angles decreased to a low value for both samples because the oxygen termination is hydrophilic. In this case, the MoS_2/O-NCD/SiO_2/Si sensor revealed properties well known for the N-type MoS_2/SiO_2/Si sensor. The response is smaller than that of traditional sensors, but a sign of change in the MoS_2 layer can still recognize the type of gas, i.e., the resistivity increases for oxidizing (NO_2) and decreases for reducing (NH_3) gas. Then, the oxidized MoS_2/O-NCD/SiO_2/Si sensor was exposed to the low-temperature H-termination process, referred to as the recovery of the H-termination MoS_2/H_{rec}-NCD heterostructure, which resulted in the gas responses observed for the initial MoS_2/H-NCD heterostructure. The surface functionalization by hydrogen was performed in a focused MW plasma CVD chamber (Aixtron P6 system, 300 sccm of H2, 10 min, 300 °C). These layers are further referred to as H_{rec}-NCD. Noticeably, samples exposed to the low temperature hydrogenation process revealed lower contact angles (<60 °) than the samples hydrogenated by the high-temperature process provided by the focused plasma system (contact angles >100 °). A lower temperature was used for recovery because higher temperature caused destruction of the wire bonding and MoS_2 active layer. Consequently, the induced surface conductivity should also be less effective, which was confirmed by the lower response of the

recovered MoS_2/H_{rec}-NCD heterostructure. Therefore, the response is lower but shows a similar course as before oxygen termination.

Table S1 Water contact angle measurements.

	MoS_2 on diamond	Reference diamond
Fabricated and measured layer<br><br>(H-termination NCD)	**106.5 °**<br>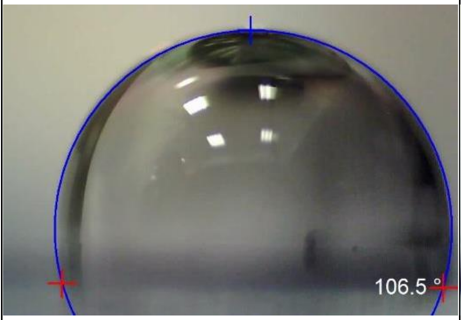	**113.8 °**<br>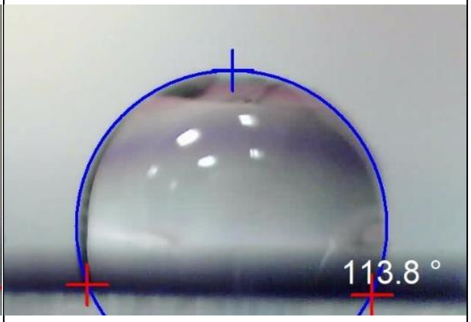
After oxygen plasma<br><br>(O-termination NCD)	**Too low**<br>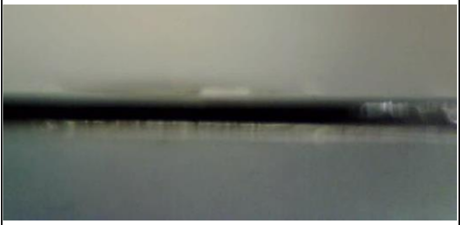	**14.4 °**<br>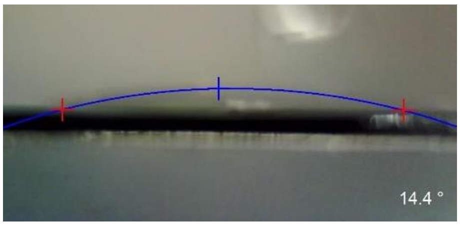
After hydrogen plasma*<br><br>(Recovery of H-termination)<br><br>* partial surface termination due to low-temperature process	**56.5 °**<br>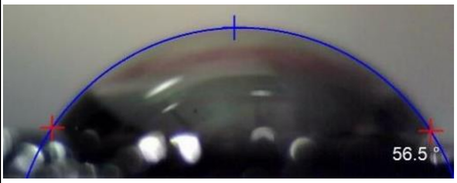	**58.9 °**<br>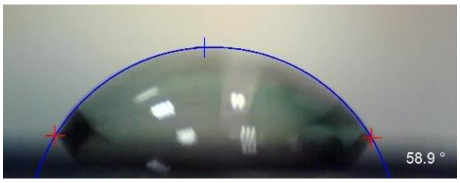

Table S2 Measured responses of MoS_2/H-NCD/SiO_2/Si and MoS_2/O-NCD/SiO_2/Si.

	MoS_2 / NCD / SiO_2 / Si heterostructure
Fabricated and measured layer<br><br>(H-termination NCD)	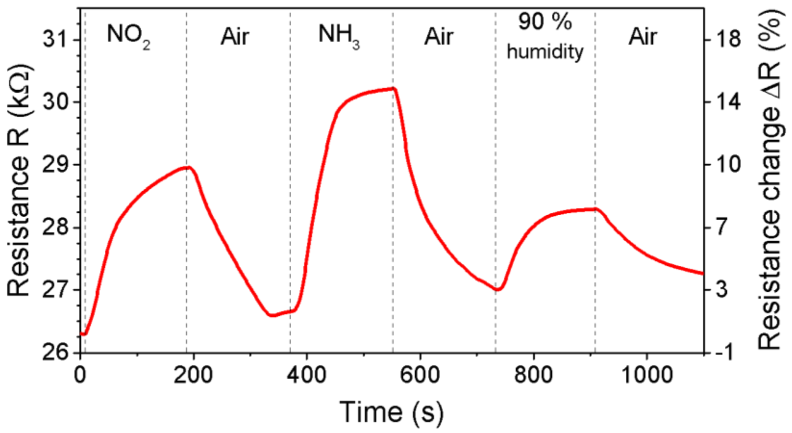
After oxygen plasma<br><br>(O-termination NCD)	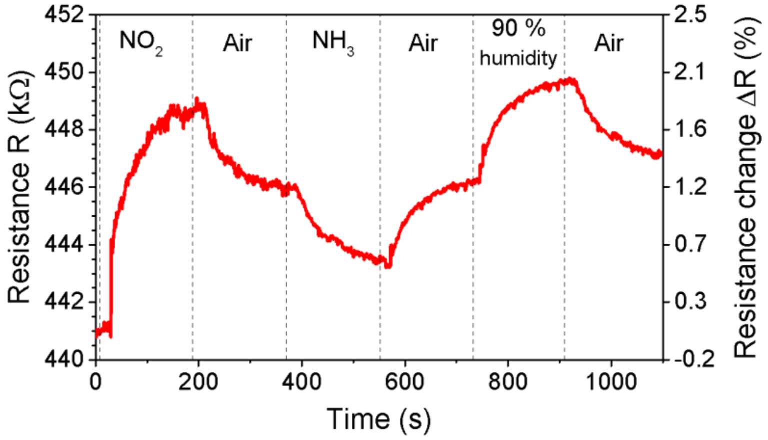
After hydrogen plasma*<br><br>(Recovery of H-termination)<br><br>* partial surface termination due to low-temperature process	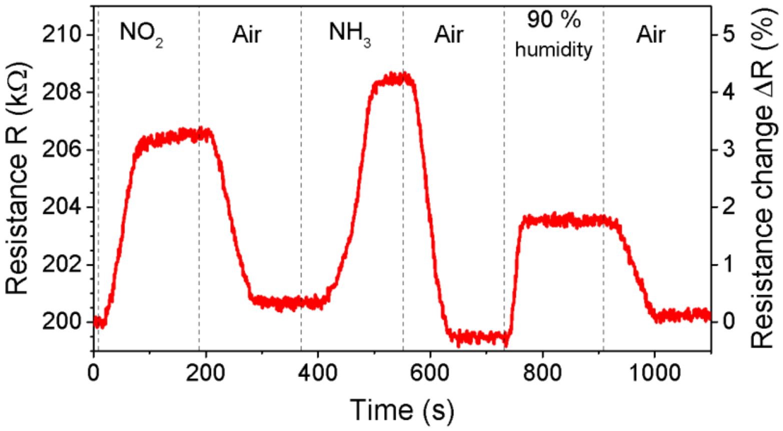
